# A Comprehensive Evaluation of *Enterobacteriaceae* Primer Sets for Analysis of Host-Associated Microbiota

**DOI:** 10.3390/pathogens11010017

**Published:** 2021-12-23

**Authors:** Carolina N. Resendiz-Nava, Hilda V. Silva-Rojas, Angel Rebollar-Alviter, Dulce M. Rivera-Pastrana, Edmundo M. Mercado-Silva, Gerardo M. Nava

**Affiliations:** 1 Facultad de Quimica, Universidad Autonoma de Queretaro, Cerro de las Campanas S/N, Queretaro 76010, Mexico; carolina.resendiz.90@gmail.com (C.N.R.-N.); dulceriverap@gmail.com (D.M.R.-P.); mercado501120@gmail.com (E.M.M.-S.); 2 Posgrado en Recursos Geneticos y Productividad, Produccion de Semillas, Colegio de Postgraduados, Km 36.5 Carretera Mexico-Texcoco, Texcoco 56230, Mexico; hsilva@colpos.mx; 3 Centro Regional Morelia, Universidad Autonoma de Chapingo, Michoacan 58170, Mexico; rebollaralviter@gmail.com

**Keywords:** Proteobacteria, *Enterobacteriaceae*, *Enterobacterales*, *16S rRNA*, primer set, taxonomic update

## Abstract

*Enterobacteriaceae* is one of the most important bacterial groups within the Proteobacteria phylum. This bacterial group includes pathogens, commensal and beneficial populations. Numerous *16S rRNA* gene PCR-based assays have been designed to analyze *Enterobacteriaceae* diversity and relative abundance, and, to the best of our knowledge, 16 primer pairs have been validated, published and used since 2003. Nonetheless, a comprehensive performance analysis of these primer sets has not yet been carried out. This information is of particular importance due to the recent taxonomic restructuration of *Enterobacteriaceae* into seven bacterial families. To overcome this lack of information, the identified collection of primer pairs (n = 16) was subjected to primer performance analysis using multiple bioinformatics tools. Herein it was revealed that, based on specificity and coverage of the *16S rRNA* gene, these 16 primer sets could be divided into different categories: *Enterobacterales-*, multi-family-, multi-genus- and *Enterobacteriaceae*-specific primers. These results highlight the impact of taxonomy changes on performance of molecular assays and data interpretation. Moreover, they underline the urgent need to revise and update the molecular tools used for molecular microbial analyses.

## 1. Introduction

Family *Enterobacteriaceae* is an important member of the Proteobacteria phylum; this bacterial group comprises numerous genera known to colonize the small and large intestine of mammals, including humans [1]. *Enterobacteriaceae* includes numerous recognized pathogens and opportunistic bacteria associated with the occurrence of enteric illnesses, urinary tract infections, sepsis and meningitis in humans [2,3,4,5]. 

Furthermore, microbial molecular analyses have linked *Enterobacteriaceae* abundance to intestinal disorders such as ulcerative colitis, irritable bowel syndrome, diverticulitis, and Crohn’s disease [6,7,8,9,10]. Furthermore, increased levels of this bacterial group have been associated with the occurrence of non-alcoholic fatty liver disease and colorectal cancer [11,12,13].

Due to its biological importance, PCR-based approaches and massive *16S rRNA* gene sequencing have been designed and implemented for analyses of diversity and abundance of the *Enterobacteriaceae* family in different environmental samples [14,15,16].

Molecular characterization of *Enterobacteriaceae* by PCR assays requires the use of taxon-specific primers; in the last eighteen years, to the best of our knowledge, 16 different primer sets have been designed, validated and published in scientific journals [15,17,18,19,20,21,22,23,24,25,26,27]. However, a comprehensive evaluation of their performance, specificity and coverage has not yet been carried out. This is of particular significance due to the taxonomic restructuration that the *Enterobacteriaceae* family underwent in 2016 [28].

After this taxonomic restructuration, the formerly *Enterobacteriaceae* group was divided into seven new families, *Budviciaceae*, *Enterobacteriaceae*, *Erwiniaceae*, *Hafniaceae*, *Morganellaceae*, *Pectobacteriaceae* and *Yersiniaceae* [28]. Importantly, this taxonomic upgrade has not been adopted in some of the recently published microbial studies [2,29,30]. Thus, the present study was designed to evaluate specificity and coverage of previously validated and published PCR primer pairs targeting specific amplification of *Enterobacteriaceae 16S rRNA* genes. The results of the present study provide a comprehensive performance analysis of different primer sets targeting *Enterobacteriaceae* for analysis of host-associated microbiota.

## 2. Results

### 2.1. Reclassification of Formerly Enterobacteriaceae 16S rRNA Gene Sequences

**Figure 1:**
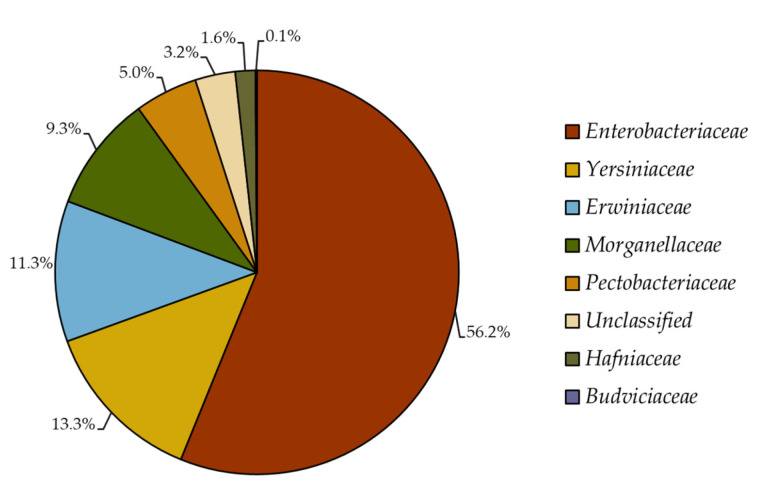
Reclassification of formerly *Enterobacteriaceae* into seven bacterial families. The *16S rRNA* gene sequences (n = 23,824) previously belonging to the *Enterobacteriaceae* family (before 2016) were retrieved from the Ribosomal Database Project and subjected to reclassification analysis using the new *Enterobacterales* taxonomy published in December 2016 [28].

The whole collection of formerly *Enterobacteriaceae 16S rRNA* gene sequences archived at the RDP database before its most recent taxonomic update (August 2020) are now classified as members of seven different families within *Enterobacterales* order. The majority (56.2%) of the sequences belong to the *Enterobacteriaceae* family, followed by *Yersiniaceae* (13.3%), *Erwiniaceae* (11.3%), *Morganellaceae* (9.3%), *Pectobacteriaceae* (5%), *Hafniaceae* (1.6%) and *Budviciaceae* (0.1%) families; interestingly, 3.2% of sequences remained unclassified at the family level (Figure 1). 

To manage taxa-associated differences across databases (i.e., LPSN (List of Prokaryotic Names with Standing in Nomenclature [31]), NCBI (National Center for Biotechnology Information [32]), RDP (Ribosomal Database Project [33]) and SILVA [34])) and to drive more accurate comparisons, a multidatabase consensus taxon list comprising 26 genera within the *Enterobacteriaceae* family was obtained (Table S1) and considered for further evaluations.

### 2.2. Identification of Primer Sets

A total of 16 primer pairs targeting *16S rRNA* genes from formerly *Enterobacteriaceae* were identified in the literature; these primers were published between 2003–2020 and were labeled *Enterobacteriaceae*-specific (Table 1). This collection of primer sets could generate PCR amplicons ranging from 49–1485 bp (based on *E. coli* numbering, accession A14565; Table 1), many of them targeting variable regions V3–V4 (18.8%), followed by V4–V5 (12.5%) (Table S2), based on *16S rRNA* variable region numbering described elsewhere [35]. For 50 percent of the primers, the authors of the manuscripts provided a detailed validation process within the *Materials and Methods* of the publication. Moreover, 31 percent of the primers were validated using at least three *Enterobacterales* genera; whereas 19 percent of the primers used DNA extracted only from *E. coli* (Table S2).

### 2.3. Primer Specificity and Coverage

Performance of 16 different primer sets (PS) was evaluated using the whole collection of *16S rRNA* gene sequences annotated and archived at the RDP. This database was chosen and used because taxonomy of *Enterobacterales* was more in agreement with the LPSN, a database regulated by the International Code of Nomenclature of Prokaryotes [31], and because RDP was more compatible with the consensus taxon list identified in the present study (Table S1). 

**Figure 2:**
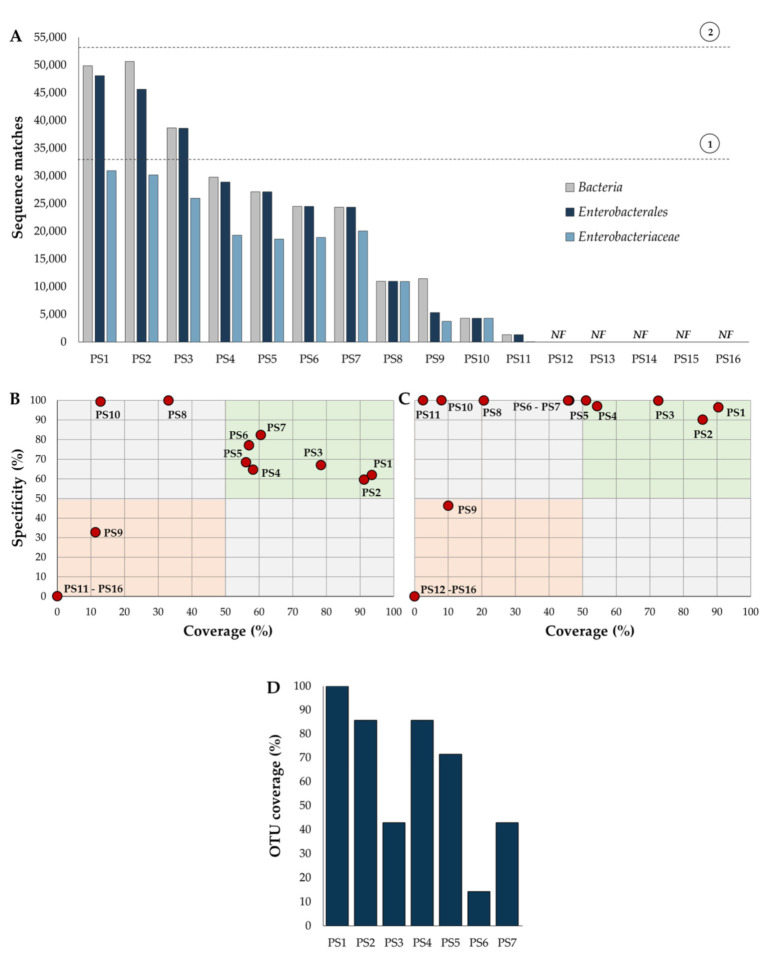
Performance of different primer sets (PS) designed for PCR amplification of formerly *Enterobacteriaceae 16S rRNA* genes. (**A**) Number of *16S rRNA* gene sequences recognized by each primer pair using the Probe Match tool at the Ribosomal Database Project (RDP). Gray dotted lines represent the total number of sequences of (1) *Enterobacteriaceae* (n = 33,092) and (2) *Enterobacterales* (n = 53,189) in the RDP. *NF*: matches not found; primer sets PS12–PS16 were unable to match sequences from the RDP database. Analysis of specificity/coverage for each primer pair at (**B**) *Enterobacteriaceae* and (**C**) *Enterobacterales* level. (**D**) OTU coverage analysis at the family level for each primer set. The seven families included in the analysis are listed in Figure 3. Because primer sets PS8–PS16 covered <50% of the genera within each bacterial family, they were not included in panel **D**. A detailed description of the OTU coverage at the genus and family levels is depicted in Table S5. Comparable results (A, B, C and D) were obtained by using the TestPrime 1.0 software at the SILVA Database (Figure S1).

Overall, the present analysis revealed that selected primer pairs varied drastically in the number of *16S rRNA* gene sequences targeted. For example, three primer pairs (PS1, PS2 and PS3) recognized >33,000 sequences, exceeding the total number (n = 33,092) of *Enterobacteriaceae* in the dataset, suggesting that these primers were out of target and cannot be considered specific for this bacterial group. Eight primers (PS4–PS11) recognized between 29,000–1300 sequences, suggesting a low coverage for *Enterobacteriaceae*. Remarkably, five primers (PS12–PS16) were unable to match sequences from the database (Figure 2A). Comparable results for all selected primer sets were obtained by using the TestPrime 1.0 software at the SILVA Database (Bremen, Germany) (Figure S1 and Table S3). Because primer pairs PS12–PS16 were unable to recognize *16S rRNA* sequences from the RDP and SILVA databases, they were not considered for further analyses. 

It is noteworthy that a preliminary assessment of potential problems associated with PS12–PS16 primer sets revealed two types of issues: (i) primer sets containing numerous sequence mismatches, and (ii) primer sets targeting the same strand of the DNA sequence (Table S4). Additional analyses are required to corroborate these observations and additional experiments should be performed to validate a corrected version of these PCR primers. The selected collection of primer pairs recognized a highly variable number of *16S rRNA* gene sequences at the order and family level. For instance, only three primer sets (PS1–PS3) recognized >70% of the *Enterobacterales* sequences, and four primers (PS4–PS7) matched >55% of the *Enterobacteriaceae* genes (Figure 2A). These results suggest that the majority of the selected primer pairs have important differences in their performance.

To corroborate this idea, a comparison of primer specificity/coverage was performed. Only seven primer sets (PS1–PS7) showed >50% specificity and >50% coverage at the family level; however, none of them reached ≥75% specificity and coverage levels—the percentage considered as acceptable in molecular microbiology [36,37,38] (Figure 2B). These results suggest that currently available primer pairs underestimate diversity and relative abundance of the *Enterobacteriaceae* family. 

As a result of the taxonomic restructuration of *Enterobacterales* [28], in the present analysis it was revealed that some of these primer sets were also unsuitable for characterization of the *Enterobacterales* order. For example, primer specificity of PS4–PS8, PS10 and PS11 were nearly 100%, but these primers had a coverage <60%. Importantly, it was identified that PS1, PS2 and PS3, had a specificity and coverage greater than 70%, suggesting that these primer pairs could be considered potential candidates for molecular analyses of the *Enterobacterales* order (Figure 2C).

**Figure 3:**
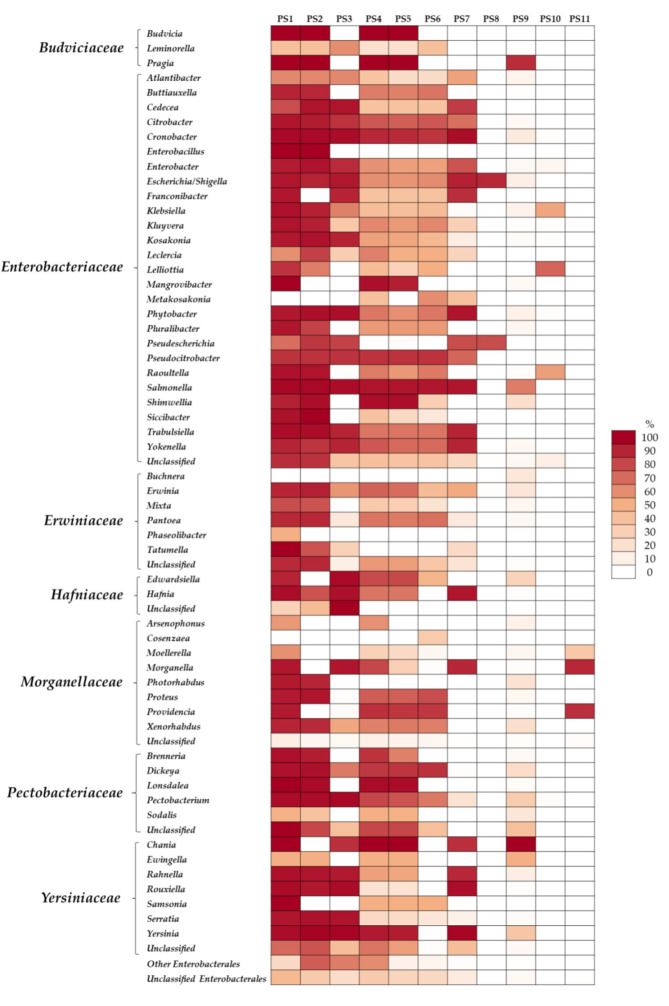
Analysis of family and genus coverage of different primer sets (PS) targeting *16S rRNA* genes. Heat map depicts sequence coverage for each taxon belonging to the *Enterobacterales* order, using the Probe Match at the RDP Database.

To evaluate the number of families and genera targeted by these primer pairs, an OTU coverage analysis was carried out. It was revealed that PS1, PS2 and PS4 recognized at least 80% of the taxa belonging to *Enterobacterales*. The rest of the primers had an OTU coverage of <70% (Figure 2D and Table S5). On average, the estimated coverage at the genus level for these three primer sets was 80%, 67% and 55% for PS1, PS2 and PS4, respectively; the remaining primer pairs showed a genus coverage ranging from 3% to 49% (Figure 3). Moreover, if a minimum coverage threshold of 50% is considered, as suggested by some authors [36,37,38], PS6 could be categorized as a suitable primer pair for analysis of *Enterobacteriaceae* (Table 2 and Figure 3).

## 3. Discussion

After the most recent taxonomic restructuration of *Enterobacteriaceae* (December 2016, [28]), databases such as LPSN, NCBI, RDP and SILVA are still considering different genera assigned to *Enterobacteriaceae* (37, 29, 33 and 23 genera, respectively, as of October, 2021). Comparable issues were also observed in other families within the *Enterobacterales* order (Table S1). These taxonomy discrepancies highlight the need to review and update databases and bioinformatics tools used for *16S rRNA* gene analyses. Moreover, researchers should revise and consider these taxonomic updates to drive more accurate conclusions when microbial analyses are performed. Unfortunately, various and recent studies are still using the formerly *Enterobacteriaceae* taxonomy removed in 2016 (for example [2,29,30,39]).

Recent studies describing the use of these 16 primers pairs (for example [40,41,42,43,44]) have not considered the latest taxonomic restructuration published in 2016. Thus, it becomes essential to review and update the specificity and coverage of these primer pairs. The limited performance of PS3 and PS5–PS11 [17,19,20,21,22,23,24], could be explained by the fact that most of these primer sets were designed and validated before the taxonomic restructuration of *Enterobacteriaceae* in 2016 [28]. Unfortunately, PS1 was published after this year [15] without considering the taxonomic restructuration [28]. These findings highlight the importance of performing frequent in-house evaluation of PCR primers. 

Importantly, the present analysis provides a framework to review and update the specificity and coverage of primer pairs that had been previously validated, used and published in the literature. In the present study, it was identified that one primer set (PS1) could be considered *Enterobacterales*-specific, five primer pairs (PS2–PS5 and PS7) multi-family specific and four primers (PS8–PS11) multi-genus specific. Out of 16, only one primer set (PS6) could be considered suitable for analysis of *Enterobacteriaceae.*

Finally, because the formerly Enterobacteriaceae family is now restructured into seven families (Budviciaceae, Enterobacteriaceae, Erwiniaceae, Hafniaceae, Morganellaceae, Pectobacteriaceae and Yersiniaceae)—all of them members of the Enterobacterales order [28]. Primer sets PS1 and PS2 could be the most suitable option for analysis of this bacterial order. These two PCR primers could be an alternative for previously known-as (before 2016) Enterobacteriaceae-specific primers for analysis of host-associated microbiota.

## 4. Materials and Methods

### 4.1. Reclassification of Formerly Enterobacteriaceae 16S rRNA Gene Sequences

To evaluate the extent of taxonomic changes in previously characterized bacterial population data, a collection of *16S rRNA* gene sequences formerly recognized as *Enterobacteriaceae* was subjected to reclassification using the current taxonomy for this bacterial group [28]. To accomplish this goal, a total of 23,824 full-length *16S rRNA* gene sequences archived at the Ribosomal Database Project (RDP) [33] and previously identified as *Enterobacteriaceae*, before its latest update (RDP Taxonomy 18, August 2020), were used for the analysis. These sequences were subjected to sequence-clustering at 100% nucleotide identity (% ID) using the CD-HIT web-tool [44]. Representative sequences (n = 16,334) were then classified using the Naïve Bayesian Classifier tool (RDP Taxonomy 18) available at the RDP [45]. This classification tool was used for the analyses because taxonomy of *Enterobacterales* and *Enterobacteriaceae* was more in agreement with the LPSN (https://lpsn.dsmz.de/; accessed on 1 October 2021)—the most widely accepted taxonomy framework [31]—and with the consensus taxon list obtained from the RDP, SILVA, NCBI and LPSN (Table S1).

### 4.2. Identification of Primer Sets

A comprehensive literature review and evaluation was carried out to identify and integrate a collection of primer sets claiming specific amplification of formerly *Enterobacteriaceae 16S rRNA* genes. To accomplish this goal, publicly available search engines and databases such as Google (https://www.google.com/), PubMed (https://pubmed.ncbi.nlm.nih.gov/), ProbeBase (https://probebase.csb.univie.ac.at/;), ScienceDirect (https://www.sciencedirect.com/) and Scopus (https://www.scopus.com/) (all accessed on 1 December 2020) were used to identify previously validated and published primer sets. From this collection of articles forward and reverse PCR primer sequences (5′–3′), amplicon size, amplicon position, targeted *16S rRNA* gene variable region [35] and genera used for PCR validation were identified and recorded.

### 4.3. Design of Primer Set #3 (PS3)

*Enterobacteriaceae 16S rRNA* gene sequences from type-strain isolates (n = 135) were retrieved from the RDP (Release 11, Update 5). These gene sequences were subjected to sequence clustering at 100% ID using the CD-HIT web-tool [45] as described above. Representative sequences were then used for primer design and in silico analysis using FastPCR software following recommendations described elsewhere [46]. The best primer set candidate (PS3) was synthesized by Integrated DNA Technologies (www.idtdna.com; Coralville, IA, USA;) and then validated in-house, using PCR assays targeting amplification of genomic DNA obtained from six *Enterobacterales* (*Salmonella enterica*, *Escherichia coli*, *Citrobacter* sp., *Enterobacter* sp., *Klebsiella* sp. and *Serratia* sp.) and six non-*Enterobacterales* strains (*Aeromonas* sp., *Bacillus* sp., *Lactobacillus* sp., *Pseudomonas* sp., *Staphylococcus* sp. and *Stenotrophomonas* sp.) from our laboratory strain collection. Genomic DNA extraction was carried out using the Quick-gDNA commercial kit (Zymo Research, Irvine, CA, USA). A PCR gradient assay (temperature range: 58–72 °C) was performed to identify the most adequate annealing temperature. PCR reactions were performed using Phire Hot Start II DNA Polymerase (Thermo Fisher Scientific, Waltham, MA, USA) and 10 ng of purified DNA. The optimized PCR protocol consisted of an initial denaturation at 94 °C for 1 min and 35 cycles of: denaturation at 94 °C for 30 s, annealing at 58–72 °C for 20 s, extension at 72 °C for 20 s and a final extension at 72 °C for 1 minute. Specificity of the PCR was analyzed via ethidium bromide/1.5% agarose gel electrophoresis.

### 4.4. Performance of Different Primer Sets Targeting Specific Amplification of Formerly Enterobacteriaceae 16S rRNA Gene

The total number of sequence matches (hits), target specificity and taxa coverage were estimated using Probe Match software, hosted at the RDP [33], for each primer pair. For these analyses, the whole *16S rRNA* gene sequence database (n = 3,356,809 sequences, Release 11, Update 5 and Taxonomy 18; as of December 2020) was used with the following parameters: *type* and *non-type strains*, *uncultured* and *isolate source*, *size* (bp) ≥1200, *good quality* and zero differences allowed. Notably, this release of the RDP comprised 53,189 gene sequences of the *Enterobacterales* order. In the analyses, total sequence matches represent the number of hits obtained from the whole RDP database. Specificity denotes the number of targeted hits divided by the total number of *Bacteria* matches. Taxa coverage corresponds to the number of targeted hits divided by the available number of taxon-specific (*Enterobacterales* or *Enterobacteriaceae*) sequences. Operational Taxonomic Unit (OTU) coverage depicts the number of families recognized by the primer set. A family was considered covered when ≥50% of the sequences belonging to that taxon were targeted by the primer set [36. Performance of 16 primers was also corroborated using the TestPrime 1.0 software, hosted at the SILVA Database (Release 138.1, n = 2,224,740 sequences; as of December 2020) [34].

### 4.5. Identification of Bacterial Groups Targeted by Previously Validated and Published PCR Primer Sets

To define the specificity of previously validated primer sets after the taxonomic restructuration of the formerly *Enterobacteriaceae* family, an OTU coverage analysis was carried out as described above. Specificity of primer sets was defined at the order, family, or genus level. To portray a detailed description of OTU coverage, a heat map was constructed showing taxon coverage accomplished for each primer. 

## 5. Conclusions

The most recent taxonomic restructuration of *Enterobacterales* has significantly impacted the ecological and epidemiological interpretation of results describing distribution and biology of this bacterial group. These taxonomic changes have also modified the specificity and coverage of previously published PCR assays designed for analysis of *Enterobacteriaceae*. Herein it is shown that only one of the currently published primer pairs could be considered suitable for an accurate and comprehensive analysis of the *Enterobacteriaceae* family. These findings highlight the imperative need to reevaluate the performance of PCR-based molecular assays designed to analyze microbial populations in human, animal and plant samples. 

## Figures and Tables

**Table 1:** List of previously validated and published primer sets designed for PCR amplification of formerly *Enterobacteriaceae 16S rRNA* genes.

Primer Set	Original Name	Sequence (5′-3′)	Amplicon Size (bp) ^b^	Position ^c^	Reference
PS1 ^a^	Forward	GGGGATAACYACTGGAAACGGTRGC	236	144–379	[15]
	Reverse	GCATGGCTGCATCAGGSTTKC			
PS2	Forward	CGTTACYCGCAGAAGAAGCA	259	482–740	[17]
	Reverse	CTGAGCGTCAGTCTTYGTCC			
PS3	Entero353-F	GCAGTGGGGAATATTGCA	474	353–826	This study
	Entero809-R	AAGGGCACAACCTCCAA			
PS4	F-ent	ATGGCTGTCGTCAGCTCGT	363	1054–1416	[18]
	R-ent	CCTACTTCTTTTGCAACCCACTC			
PS5	Entero-F234	GATGWRCCCRKATGGGA	1198	226–1423	[19]
	Entero-R1423	AKCTAMCTRCTTCTTTTGCAA			
PS6	EnterobactDmod2F	GACCTCGCGAGAGCA	161	1259–1419	[20]
	Enter1432mod	CCTACTTCTTTTGCAACCCA			
PS7	515F	GTGCCAGCMGCCGCGGTAA	312	514–825	[21]
	826R	GCCTCAAGGGCACAACCTCCAAG			
PS8	Eco1457F	CATTGACGTTACCCGCAGAAGAAGC	170	476–645	[22]
	Eco1652R	CTCTACGAGACTCAAGCTTGC			
PS9	fd2	AGAGTTTGATCATGGCTCAG	1485	7–1491	[23]
	rp1	ACGGTTACCTTGTTACGACTT			
PS10	Forward	GCGGTAGCACAGAGAGCTT	49	65–113	[24]
	Reverse	GGCAGTTTCCCAGACATTACTCA			
PS11	Forward 2	CGTTACCGACAGAAGAAGCA	259	482–740	[17]
	Reverse	CTGAGCGTCAGTCTTYGTCC			
PS12	ENT-F	GTTGTAAAGCACTTTCAGTGGTGAGGAAGG	*NF* ^d^	*NF*	[25]
	ENT-R	GCCTCAAGGGCACAACCTCCAAG			
PS13	DG74f	AGGAGGTGATCCAACCGCA	*NF*	*NF*	[23]
	RW01r	AACTGGAGGAGGGTGGGGAT			
PS14	Ent 1113	TGGCAACAAAGGATAAGG	*NF*	*NF*	[26]
	Ent 1418	CTTTTGCAACCCACT			
PS15	LUX—F	CGGTGTACCCGCAGAAGAAGCACG	*NF*	*NF*	[27]
	LUX—R	GCTTGCACCCTCCGTATTACC			
PS16	LUX—F	CGGTGTACCCGCAGAAGAAGCACG	*NF*	*NF*	[27]
	ENT—R	GCCTCAAGGGCACAACCTCCAAG			

^a^ Primer Set (PS) code assigned in the present study. ^b^ Amplicon size; ^c^ forward and reverse primer binding positions using the *Escherichia coli 16S rRNA* gene as reference (accession number A14565). ^d^ Not found.

**Table 2:** Revised list of bacterial targets accomplished by previously validated and published PCR primer sets.

Primer Set	Targeted Taxa (Coverage) ^a^
PS1	*Enterobacterales* (90%)
PS2	*Budviciaceae* (67%) + *Enterobacteriaceae* (89%) + *Erwiniaceae* (67%) + *Hafniaceae* (50%) + *Pectobacteriaceae* (80%) + *Yersiniaceae* (71%)
PS3	*Enterobacteriaceae* (58%) + *Hafniaceae* (100%) + *Yersiniaceae* (71%)
PS4	*Budviciaceae* (67%) + *Enterobacteriaceae* (65%) + *Hafniaceae* (100%) + *Morganellaceae* (63%) + *Pectobacteriaceae* (100%) + *Yersiniaceae* (71%)
PS5	*Budviciaceae* (67%) + *Enterobacteriaceae* (65%) + *Hafniaceae* (100%) + *Pectobacteriaceae* (80%) + *Yersiniaceae* (71%)
PS6	*Enterobacteriaceae* (62%)
PS7	*Enterobacteriaceae* (50%) + *Hafniaceae* (50%) + *Yersiniaceae* (57%)
PS8	*Escherichia*/*Shigella* (89%) + *Pseudescherichia* (78%)
PS9	*Pragia* (87%) + *Salmonella* (65%) + *Chania* (100%) + *Ewingella* (50%)
PS10	*Klebsiella* (53%) + *Lelliottia* (71%) + *Raoultella* (55%)
PS11	*Morganella* (89%) + *Providencia* (87%)

^a^ Primer set covered the seven families assigned to the *Enterobacterales* order. A taxon was included in this table when ≥50% of the sequences belonging to that taxon were targeted by the primer set.

## Data Availability

CD-HIT: http://weizhongli-lab.org/cd-hit/; List of Prokaryotic Names with Standing in Nomenclature: https://lpsn.dsmz.de/; NCBI Taxonomy: https://www.ncbi.nlm.nih.gov/taxonomy; Ribosomal Database Project: https://rdp.cme.msu.edu/; SILVA: https://www.arb-silva.de/ (all accessed on 1 December 2020).

## Supplementary Materials

The following are available online at https://www.mdpi.com/article/10.3390/pathogens11010017/s1: Table S1: *Enterobacterales* families and genera included in the LPSN, NCBI, RPD and SILVA databases (as of October 2021); Table S2: List of previously validated and published primer sets designed for PCR amplification of formerly *Enterobacteriaceae 16S rRNA* genes; Table S3: OTU coverage analysis, at the family level, using the SILVA database; Table S4: Primer sets PS12–PS16 and potential problems; Table S5: OTU coverage analysis, at the family level, using the RDP database; Figure S1: Performance of different primer sets (PS) designed for PCR amplification of formerly *Enterobacteriaceae 16S rRNA* genes using the TestPrime 1.0 software at the SILVA Database; Figure S2: Analysis of family and genus coverage of different primer sets (PS) targeting *16S rRNA genes* using the TestPrime 1.0 software at the SILVA Database.
