# Supplementary material for: A Comprehensive Evaluation of Enterobacteriaceae Primer Sets for Analysis of Host-Associated Microbiota
Source: Pathogens. 2021 Dec 23;11(1):17. doi: 10.3390/pathogens11010017 (PMC8780275; doi:10.3390/pathogens11010017)
Supplement: Supplementary file 1 [file pathogens-11-00017-s001.zip › figures.v3-proofed.pdf]

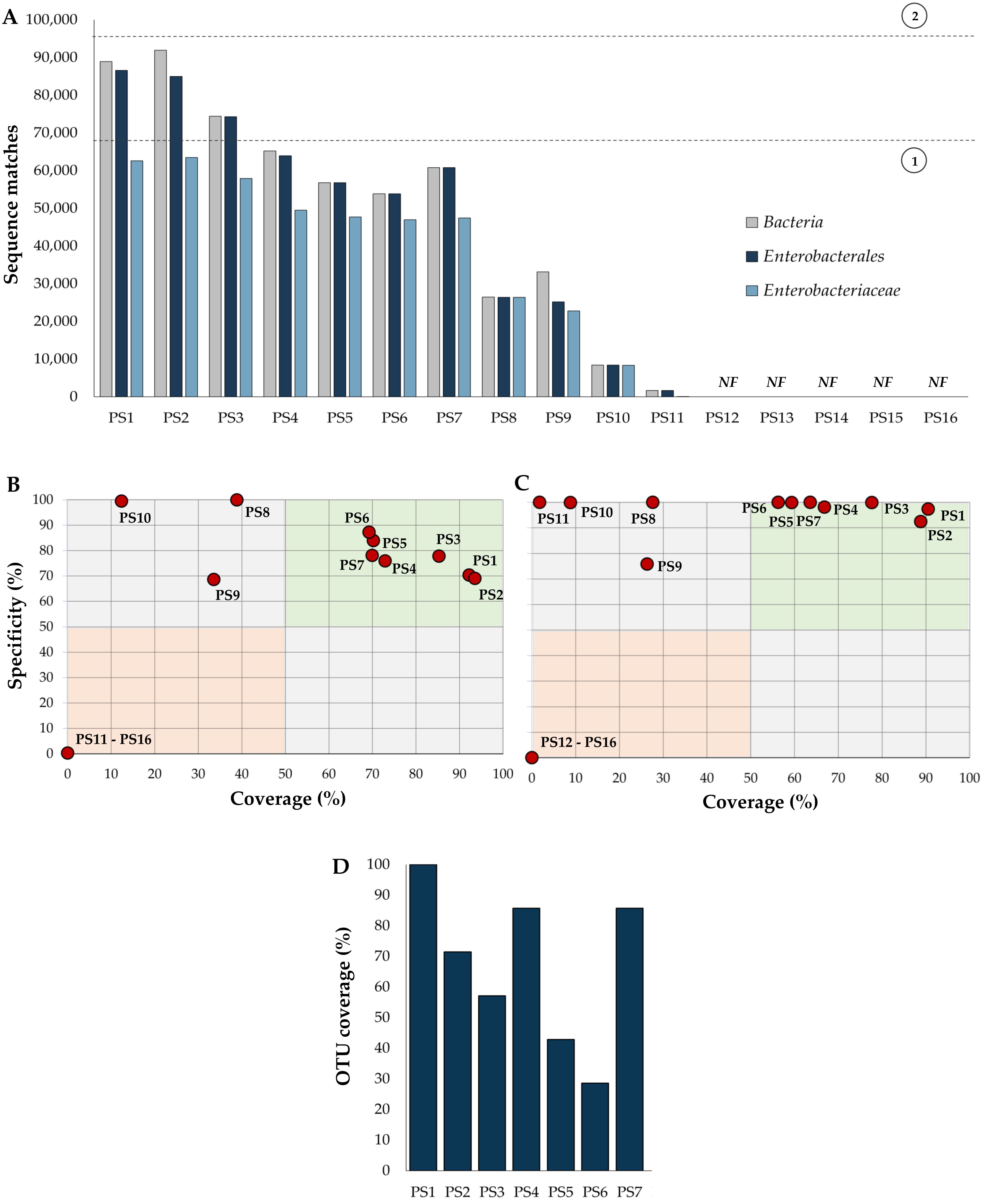

Figure S1. Performance of different primer sets (PS) designed for PCR amplification of formerly-*Enterobacteriaceae 16S rRNA* genes. **A)** Number of *16S rRNA* gene sequences recognized by each primer pair using the TestPrime 1.0 software at the SILVA Database. Gray dotted lines represent the total number of sequences of **1)** *Enterobacteriaceae* (n = 67,849) and **2**) *Enterobacterales* (n = 95,655) in the SILVA database. NF: matches not found; Primer sets PS12 - PS16 were unable to match sequences from the SILVA database. Analysis of specificity/coverage for each primer pair at **B)** *Enterobacteriaceae* and **C)** *Enterobacterales* level. **D)** OTU coverage analysis at the family level for each primer set. The seven families included in the analysis are listed in Figure 3. Because primer sets PS8 - PS16 covered <50% of the genera within each bacterial family, they were not included in panel **D**. A detailed description of the OUT coverage at the genus and family levels are depicted in **Table S4**.

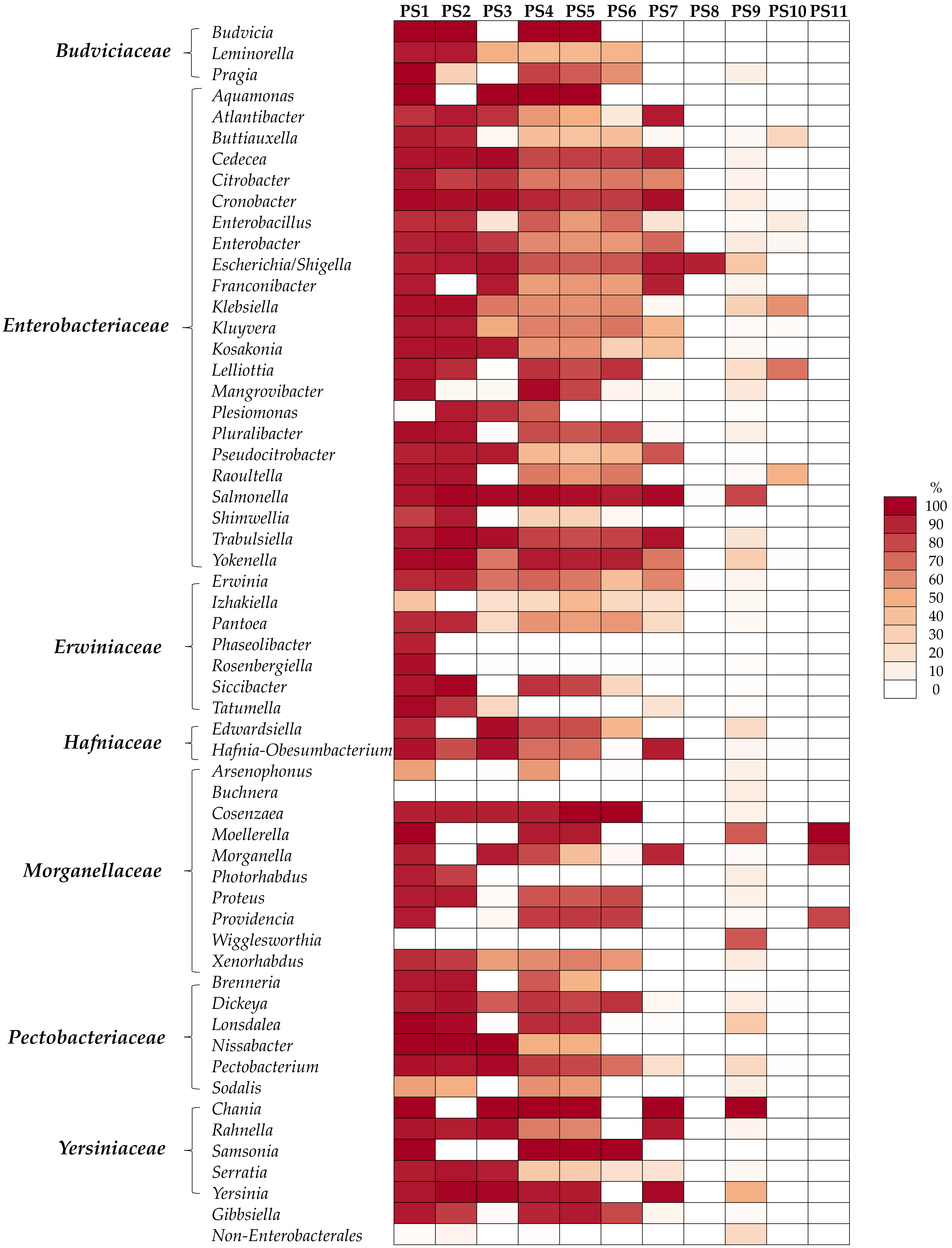

Figure S2. Analysis of family and genus coverage of different primer sets (PS) targeting *16S rRNA* genes. Heat map depicts sequence coverage for each taxon belonging to the *Enterobacterales* order, using the TestPrime 1.0 software at the SILVA Database
